# The Psychological Impact of Strict and Prolonged Confinement on Business Students during the COVID-19 Pandemic at a Spanish University

**DOI:** 10.3390/ijerph18041710

**Published:** 2021-02-10

**Authors:** Anne Marie Garvey, Inmaculada Jimeno García, Sara Helena Otal Franco, Carlos Mir Fernández

**Affiliations:** Social Dimension and COVID-19, Interdisciplinary Research, Department of Economics and Management, Universidad de Alcalá, 28802 Madrid, Spain; inmaculada.jimeno@uah.es (I.J.G.); sarah.otal@uah.es (S.H.O.F.); carlos.mir@uah.es (C.M.F.)

**Keywords:** anxiety, COVID-19, confinement, GAD-7, global health, mental health, social support, sleep disorder, students

## Abstract

The study was carried out to examine the situation of university students from one month after the beginning of a very strict confinement process in Spain during the COVID-19 pandemic. Students responded to a survey which included the 7-item Generalized Anxiety Disorder Scale (GAD-7) together with other questions relating to their general well-being from the European Quality of Life Survey (EQLS). A total of 198 university students answered the web-based survey. The questionnaire was generated using Microsoft Forms and was explained and distributed online. The results indicated that around 18.7% of students were suffering from severe anxiety and 70.2% were suffering either mild or moderate anxiety at this point of the strict confinement process. The findings show that when emotional well-being (quality of sleep, the perception of feeling fear, death of a relative) is reduced and material well-being is negatively affected (income level) anxiety levels are increased. On the other hand, the results show that having good interpersonal relationships with family members and taking care of personal development (routines and habits that make them feel good) help reduce anxiety levels. The female students in the sample also suffered higher levels of anxiety than males during strict confinement.

## 1. Introduction

The novel coronavirus (COVID-19) was first detected in Wuhan (Hubei Province, China) in December 2019. The virus spread rapidly all over China and to other countries having a substantial impact on the lives of people around the globe. The mounting cases and deaths have posed major public health and governance challenges [1]. The World Health Organization (WHO) [2] declared a global pandemic in a media briefing in the second week of March 2020. They advised countries to strike a fine balance between protecting health, minimizing economic and social disruption, and respecting human rights. Although WHO’s mandate is public health, they commented that the crisis would touch every sector.

In Spain, a nationwide state of alert was declared on 14 March 2020 by the Spanish government through the publication of Real Decreto 463/2020 [3] and a population lockdown began on 16 March 2020 schools and universities were progressively closed in the week starting 9 March and online learning was the medium of education from 16 March 2020. The strict conditions of confinement that affected the country were the prohibition of leaving one’s place of residence with certain exceptions. These exceptions included the acquisition of food, pharmaceuticals and necessities, health centre visits, displacement to the workplace and returning to habitual residence, assisting dependent and vulnerable individuals, travel to financial entities, force majeure and other similar activities. The state of alarm continued until the 21 June 2020, although there was a gradual lifting of restrictions from 2 May 2020.

Adverse mental health problems began to be detected by the population in general due to the situation [4]. Mental health consequences were predicted [4] and included distress reactions such as insomnia, anger, extreme fear of illness even in those not exposed and mental health disorders including post-traumatic stress disorder, anxiety disorders, depression, and somatization. The negative impact on the mental health of university students is expected to be high [5]. Anxiety will grow extensively and will be exacerbated due to uncertainties and intensification of the information flow [5]. Previous research shows that individuals experienced negative emotional responses, such as anxiety and depression symptoms during an outbreak [6]. Students have been detected as a vulnerable group, experiencing significant levels of anxiety, and proposals have been made for reducing stress in university students [7].

At the Economics, Business and Tourism Faculty at the University of Alcalá, emotional and mental health problems began to be noticed by educators. It was considered important to examine the impact that the strict confinement restrictions were having on this community to have a more informed report on the situation and to mitigate the adverse effects on this community. This research was carried out to measure the effect that a decrease in students’ quality of life has on anxiety levels using the anxiety measurement scale GAD-7 and to observe factors which had a dependent relation on anxiety levels. These factors included questions relating to emotional well-being, interpersonal relationships, material well-being and personal development.

At this stage, we advance the results of this research study to highlight that the surveyed students were suffering from abnormal anxiety (89.9%) between one and two months after strict confinement began. Females suffered more anxiety in general than males and also, severe anxiety was significantly higher in the female gender (21.8% as opposed to 12.31% for males). Certain factors were associated with anxiety risk including perception of being negatively affected by confinement, income negatively affected, and the perception of feeling fear. Other factors were associated with protection against anxiety including a positive relation with family members, perception of feeling calm and relaxed, looking after personal development by following daily habits and satisfactory sleep quality.

## 2. Objectives and Methods

### 2.1. Study Sample

The target participants comprised 366 undergraduates. These participants are students from accounting and business subjects at the Faculty of Economics, Business and Tourism. A web-based survey using Microsoft Forms was announced to students and participation was voluntary. We obtained 198 usable responses. This represents a 54% response rate. Students received detailed information of the purpose of the study and they provided their online consent before proceeding with the study.

### 2.2. Survey Instrument

The survey was prepared through Microsoft Forms and consisted of 14 questions. The questions included some introductory demography questions, the GAD-7 matters and a series of issues to analyse some of the factors that are part of people’s quality of life including sleep quality and how the virus is affecting personal emotions. For this reason, questions relating to the dimension of emotional well-being, interpersonal relationships, material well-being, and personal development have been included. These questions allowed us to analyse whether there is a close association with these aspects of quality of life and GAD-7. The survey was distributed among students from one month after strict confinement. The survey consisted of three areas: (1) demographic data; (2) well-being and quality of life; (3) mental health status. Demographic data were collected on gender, academic information, living conditions and income status during the pandemic.

The quality of life survey has been structured in line with the definition made by Verdugo et al. [8]. These authors state that the quality of life of a person is composed of eight dimensions (emotional well-being, interpersonal relationships, material well-being, personal development, physical well-being, self-determination, social inclusion and rights). This study analyses the relationship that some of these dimensions (emotional well-being, interpersonal relationships, material well-being, personal development and physical well-being) have with anxiety during the pandemic. Self-determination, equality rights and social inclusion are not analysed because they are considered to be factors already satisfied by the respondents.

The well-being and quality of life questions used are taken from the European Quality of Life Survey (EQLS). It is a cross-sectional survey carried out by the European Foundation. The EQLS examines the different dimensions in which quality of life is composed.

The 7-item Generalized Anxiety Disorder Scale (GAD-7) was used as part of the survey. This scale is one of the most widely used. It takes only a few minutes to complete and scoring is easy [9]. It can be applied for screening, diagnosis, and assessing the severity of anxiety disorders [10]. It is a suitable measure in clinical practice due to its efficiency and reliability for diagnosis [11]. The well-known GAD-7 scale includes seven elements based on seven core symptoms; the respondents reply as to how often they were troubled by each symptom during the previous two-week period [12]. Scores of 5, 10, and 15 represent cut-off-points for mild, moderate, and severe anxiety, respectively. The GAD-7 score is calculated by assigning scores of 0, 1, 2, and 3, to the response categories of “not at all”, “several days”, “more than half the days”, and “nearly every day”, respectively. The scores are summed for the seven questions.

### 2.3. Literature Review

Several studies have been published, to date, in relation to the level of anxiety of students during lockdown for COVID-19. These studies are carried out at different stages of lockdown and with different confinement conditions. Cao’s [13] was one of the first studies to be carried out and published on anxiety in students during the COVID-19 pandemic. The sample was made up of 7143 Chinese college students who live in the Hubei Province, two thirds of which were female. They used a GAD-7 scale for measuring anxiety and a survey format for associated questions. It is not clear from the study the exact dates of when it was carried out (we estimate between 23 February 2020 and 14 March 2020 or the conditions or length of confinement. Over three quarters of the students had no symptoms of anxiety while the proportion of students with mild, moderate and severe anxiety were 21.3%, 2.7% and 0.9%, respectively. There was no significant difference in the results between males and females. Another study by Bourion-Bedes et al. [14] was on university students from Nancy in the Grand Est region of France. This region was especially affected by the virus and had confinement restrictions. An online survey was used and there were 3936 respondents from 7 May until 17 May 2020. The GAD-7 scale was also used for measuring anxiety. The results showed that students suffered normal, mild, moderate and severe anxiety in the following percentages of 38.9%, 36%, 15.2% and 9.8%, respectively. Females showed higher anxiety levels than males and another risk factor for higher levels of anxiety was having a relative or acquaintance from their housing hospitalised for COVID-19. A third research paper by Patwary et al. [15] involving 544 respondents from several universities in Bangladesh also showed high anxiety levels during lockdown. This research paper also used GAD-7 as a research instrument through an online survey. The results showed that 78.2% of the sample were suffering from abnormal anxiety and 20.8% had severe anxiety. Once again, significantly higher levels of severe anxiety were detected in the female gender. This survey was completed by students three weeks into strict lockdown conditions. A separate study by Islam et al. [16] incorporating 476 university students living in Bangladesh as respondents, showed slightly higher levels of anxiety than the previous case examined. This study was carried out after a longer period of strict lockdown (between six and seven weeks after strict lockdown began). The results show normal, mild, moderate and severe anxiety with percentages of 18.3%, 38.9%, 24.8% and 18.1%, respectively. In a similar study by Odriozola-González et al. [17] on Spanish university students involving a sample of 2530 during the first weeks of strict lockdown, showed 21.34% of moderate to severe (inclusive) anxiety levels. The research instrument used here was DASS-21. Finally, we examined a study including a sample of Turkish university students during lockdown by Aslan et al. [18]. The sample included 358 participants and GAD-7 was used as one of the research instruments in the study. Although the exact time of the survey after initial lockdown is not indicated with precision, it would seem to be after at least one month after the start of confinement. Turkey also took confinement measures but would seem to be less strict than those of Spain, for example. The anxiety levels reported by GAD-7 are 12.57%, 35.75%, 28.77% and 22.91% for normal, mild, moderate and severe anxiety, respectively. Females also showed higher anxiety levels than males.

### 2.4. Data Analysis

Data were analysed with SPSS Version 26. Firstly, a descriptive analysis of the data was carried out. A univariant analysis was used to determinate the univariate associations between the variables with GAD-7. The relevant factors chosen for inclusion in the full model are those which showed a significant association with GAD-7. A multivariate regression analysis was used to determine the variables associated with severe anxiety using GAD-7 score [12]. The stepwise selection system was used to determine the full model. The goodness of fit was assessed by calculating the model determination coefficient (R^2^), and the Akaike test (AIC) allowed the selection of the best model.

## 3. Results

Demographic and selected characteristics of the participants in the study are included in Table 1. From the sample, 67.2% were female and the remaining 32.8% were male. All were studying a business-related degree course and 58.08% were second-year students. Over 62.6% of respondents mentioned that their immediate family’s economy had been negatively affected and 94.4% of students were living with their family during the confinement period. Of the sample, 8.6% had experienced the death of a family member and 6.6% had lost a friend to the virus.

### 3.1. Anxiety Levels among Students from One to Two Months after Strict Confinement

Table 2 shows anxiety levels suffered by students after being strictly confined from one to two months. Normal levels of anxiety were found for 11.1%; this rose to 21.53% in the case of the male sample and decreased to 6.01% for females. Over 89% of the sample showed anxiety of some sort and 18.7% showed severe anxiety. The level of severe anxiety was significantly higher among females at 21.8% in comparison with 12.31% for males (see Table 3). Higher levels of anxiety in females have been detected in previous studies [14,15,16,18].

Even though 55.2% of the sample (Table 2) show moderate and severe levels of anxiety, this did not prevent them from carrying out their everyday tasks (work, doing things at home and social relations). Table 4 shows that 66.7% had no difficulty or some difficulty doing their everyday tasks; however, 33.3% found it very difficult to continue with them. All individuals suffering from severe anxiety had some sort of difficulty completing everyday tasks.

The results of the univariant analysis are reported in Table 5. The results from the univariant test will be explained in the next sections.

(a) Univariant Analysis

### 3.2. Anxiety Levels in Students Who Perceived They Were Negatively Affected by the State of Alarm Due to COVID-19 (Confinement)

There is a direct relationship between anxiety level and how students perceived they had been affected by the state of alarm and confinement (see Table 5 and Table S1). Those students who considered they were affected a lot by the circumstances showed that 30.1% of them suffered severe anxiety, 33.33% endured moderate anxiety and 28.5% experienced mild anxiety. This contrasts with the overall results which showed 18.7% with severe anxiety thus showing that the more negatively the circumstances were self-perceived increased the severe anxiety level and lowered the levels of mild and moderate anxiety.

Students who considered they were not negatively impacted by the circumstances showed that 50% were not suffering from any form of anxiety. This is a very different result to what we find in the overall group of respondents which showed 88.9% were showing some sort of anxiety.

Therefore, the more affected the student self-perceived their situation to be, the higher the levels of severe anxiety, and in students who did not self-perceive to be affected by the situation, the levels of anxiety were greatly reduced.

### 3.3. Anxiety Levels in Students Whose Total Immediate Family Income (Parents, Siblings) Was Negatively Affected during Confinement for COVID-19

Just over 62.6% of students said that the family income had been negatively affected during confinement. As expected, this group experienced higher levels of severe anxiety (21.7%) while 8.87% of this set were unaffected. Increased levels of anxiety in relation to income loss were found among students in employment rather than by immediate family members [19] (see Table S2).

In the case of students’ families whose income was not negatively impacted, a lower intensity of severe anxiety was detected (13.51%). Additionally, 14.86% of this same group did not show any signs of anxiety, which is a higher percentage than that observed for the total sample.

### 3.4. Anxiety Levels in Students Who Perceived a Feeling of Fear

This question was asked to reflect on their feeling of fear (perception of fear) at that point of lockdown. The issue did not relate to any specific fear but to a general sense of fear to the situation in general. There was a lot of uncertainty and rapid changes (increasing number of deaths and infected individuals, uncertainty as to how one could become infected, adaptation to online learning, worry about evaluations, etc.) at that time. Spain was very severely affected by the virus and confinement was very strict at the time of the survey.

Around 14.14% of students expressed that they never perceived the feeling of fear. The main answer was that they perceived fear less than 50% of the time (48.98%). Almost 2.02% expressed that they always felt fear at this point of confinement, and we observe a relation with anxiety levels from the surveyed students. This is the perception of fear by the respondent which, of course, is related to anxiety, however, fear is not expressly tested using scientific measurements. Additionally, 75% were suffering from severe anxiety and all were experiencing either severe or moderate anxiety (see Table S3).

We conclude, therefore, that there is a direct relation between the perception of feeling fear and anxiety levels in students at this stage of confinement.

### 3.5. Anxiety Levels in Students Who Had Their Sleep Patterns Affected Negatively

Many students experienced some effect on their sleep patterns (87.88%). However, 25.25% had disimproved sleep quality all the time and 40% of them were suffering from severe anxiety. Only 12.12% said that their sleep was unaffected, and no severe anxiety was found, with 37.5% not suffering any type of anxiety at all. Mild levels were at 37.5% and moderate levels at almost 25% (see Table S4).

Of the 15.15% that claimed their sleep was a little bit affected showed no signs of severe anxiety and 26.6% were not suffering any anxiety symptoms at all. This shows that individuals have higher anxiety levels associated with disimproved sleep quality.

### 3.6. Anxiety Levels in Students and Their Relationship with Their Family

Previous studies have shown that satisfaction in family relationships has a direct effect on anxiety levels [13]. This direct effect is reaffirmed in the study (*p*-value < 0.001). Therefore, students who expressed to be very satisfied or completely satisfied with their family totalled 70.7% of the total sample. The severe anxiety level for this group was 14.28%, a lower rate than 18.7% from the full sample. Almost 12.14% of this group were not experiencing any type of anxiety which is slightly higher than the result (11.1%) for the global sample (see Table S5).

Students who expressed that they were dissatisfied (9% of all respondents) with their family relationships were showing severe levels of anxiety (50%) and those who expressed that they were somewhat satisfied (14.64% of the sample) showed 10.34% for severe anxiety.

Once again, we can see a direct relation between anxiety levels and family relations. Students who are satisfied with their family relations suffer less severe anxiety than those who do not have satisfactory relations with their family.

### 3.7. Anxiety Levels in Students Who Felt That Continuing with Their Daily Habits Helps Them during Confinement

The objective was to find a possible relation between anxiety levels and the perceived help obtained by following daily routines. We included examples of daily habits here which were online classes, physical activity at home, personal relationships online, reading, etc.

Around 77.27% of students felt that by following their normal daily habits helped them during confinement. The severe anxiety level was reduced to 15.68% and those with normal anxiety increased to 13.07%. Students who did not perceive help by following their daily habits were 12.62% of the students surveyed. All suffered some sort of anxiety and 32% suffered severe anxiety (see Table S6).

The rest of the sample (10.1%) felt indifference in relation to the help they received from being able to follow their daily tasks. From this group, 25% suffered severe anxiety and almost 10% were not suffering any anxiety. We conclude that following daily habits has an association with a reduction in anxiety suffering.

### 3.8. Anxiety Levels in Students in Relation to Whether They Feel Calm and Relaxed

Once again, we obtained similar results to the feeling of calmness and relaxation on levels of anxiety. A very small percentage (2%) said they were always calm and relaxed, and they showed no signs of moderate or severe anxiety with a high percentage (75%) experiencing no anxiety at all (see Table S7).

Those who claimed that they were never calm and relaxed (11.11%) showed very high levels of anxiety (68.18%) and all suffered from some sort of anxiety.

Just over 11.11% said that they felt calm and relaxed most of the time and they had no indications of severe anxiety with 45.4% not showing any signs of anxiety.

We can therefore confirm a direct relation between feeling more relaxed and calmer with lower levels of anxiety and vice versa.

### 3.9. Anxiety Levels in Students Who Experienced the Death of a Relative or a Friend

Less than 9% (8.58%) of students surveyed had been affected by the death of a relative. From this group, only 5.88% did not suffer any type of anxiety and the severe form was suffered by 17.64% (see Table S8). This result is lower than that calculated for the total sample. The level of moderate anxiety increased substantially in this group, however (rose to 47% from 36.4%), and there was a lower level for mild anxiety (29.41% from 33.8%).

Some students (6.56%) lost a friend to death during the confinement period. Over 15.38% showed normal anxiety levels. However, severe anxiety was far lower (7.69%) in this case in comparison to the total sample which was 18.7%. The level of moderate anxiety increased slightly in this group (from 36.4% to 38.46%).

The death of a relative from COVID-19 showed a change in anxiety intensity from mild to moderate with a slightly lower level of severe anxiety during the confinement period. The observations found for the death of a friend were different to those of a relative. More information would be necessary to draw satisfactory conclusions from this.

(b) Multivariant Analysis

The variables that resulted significantly in the univariate analysis (Table 5) are those that we used to develop the multivariate logistic regression model.

In this model, we observed that the variables that were significant when analysing the relationship between severe levels of anxiety are (see Table 6): perception of being negatively affected by strict confinement, feeling calm and relaxed, feeling fear, sleeping worse, feeling isolated, family relationships, difficulty in performing work during confinement.

More specifically, the relationship between sleeping worse and severe anxiety levels should be highlighted. It is also important to note that a direct relationship is detected between sleeping worse more than half of the time and suffering severe anxiety. In this case, we reconfirm the result found in the univariate analysis which shows that disimproved sleep patterns aggravate severe anxiety levels.

Finally, we paid special attention to the relationship between the difficulty of working during confinement and levels of anxiety. We observed that there is a direct relationship when students feel that it is a bit difficult to carry out their tasks and severe anxiety. This relationship increases when the anxiety level is moderate, meaning that a moderate level of anxiety shows an increase in the relationship of finding it a little bit difficult to perform tasks.

## 4. Discussion

The COVID-19 outbreak has provoked an important psychological impact on the population. It is the objective of our study to examine the psychological impact on business students at a Spanish University. Spain is one of the countries that was most severely affected by the pandemic during the months from March to June 2020. Most countries introduced some sort of confinement, however, the confinement conditions in Spain were especially severe. In a previous study of 129 quarantined individuals, a high level of psychological distress was discovered [20]. In this same study, the longer the period of confinement showed an increased prevalence of post-traumatic symptoms.

In particular, we examined the psychological response to strict confinement for COVID-19 on a specific sample of students at the University of Alcalá between one and two months after the introduction of the strict confinement requirements. In the literature review in Section 2.3 of this manuscript, we refer to the findings on anxiety suffering by university students during confinement for COVID-19. All studies showed high levels of anxiety in students and higher levels of severe anxiety when the survey was performed after a long and strict confinement period [16,18] Most of the studies showed higher levels of anxiety in females [14,15,16,18]. Cao et al. [13] did not find any difference in anxiety levels between females and males. Other studies showed this same pattern of females suffering higher levels of anxiety, although the participants in the sample are different. For example, Madani et al. [21] found higher levels of anxiety in females on a study of a part of the general population, and Jin et al. [22] found similar results but in a sample of students from 13 to 26 years old.

The Spanish study was carried out just two weeks after lockdown and a different scale was used and high levels of severe anxiety were discovered but lower than those for prolonged lockdown. The lowest level of severe anxiety was found in the Chinese study [13] with 0.9% suffering from severe anxiety and a much lower level in the overall sample. Finally, the study on students from an area particularly affected by the virus in France showed 9.8% severe anxiety and 61% suffering from anxiety.

Our study shows that individuals have higher anxiety levels associated with disimproved sleep quality. These findings are in line with other preliminary evidence obtained which suggests that anxiety is a common psychological reaction to the COVID-19 pandemic and that it may be associated with disturbed sleep [23]. A decrease in the quality of sleep was evidenced by Greek students [24].

Patwary [16] also discovered that post graduate students were more likely to suffer from mental illness during the pandemic and that financial hardship, academic disruption, and worry about the health of family members were closely associated with anxiety levels. In the study by Odriozola-Gonzalez [17], students from the Social Sciences and Law Faculty showed the highest level of extremely severe anxiety at over 10%.

Other reasons for emotional disorder have also been studied, for example, Lambert et al. [25] explained that the lack of adequate exposure to sunlight due to prolonged confinement could result in decreasing levels of serotonin in the body and contribute to emotional disorders. Some studies on confinement have found that students in quarantine for less time reported more positive psychological behaviour than those confined for longer periods of time, for example, Brooks et al. [26]; Gritsenko et al. [27]; Pan et al. [28].

Our study has some limitations including a lack of knowledge on pre-existing mental illness of students and also, an online survey could be associated with response bias or self-selection bias. It is also important that the GAD-7 scale is tested at one point in time and would need to be assessed several times for a diagnosis to be made, i.e., it is a measure of anxiety levels at a particular point in time and not a diagnosis of an anxiety illness.

## 5. Conclusions

This study shows that students at a Spanish university studying business-related careers suffered from abnormal anxiety (89.9%) during strict confinement after one month and up to two months of very strict confinement. Females were discovered to suffer higher levels of anxiety (95%); the percentage of females suffering from severe anxiety rose to 21.8% as against 12.31% in the male respondents.

There was a direct relation between levels of anxiety and individuals who perceived that they were negatively affected due to confinement or those whose family income decreased. There was also an unequivocal effect on anxiety levels when comparing with certain emotions including perceiving the feeling of fear or being calm and relaxed. The relation that students had with their family was important in relation to the impact on anxiety levels, as was being able to continue with daily habits and sleep quality.

Although few observational studies are published at present, the long-term mental health impact of COVID-19 may take some time to become fully apparent, and managing this impact requires significant effort by the health care system and by psychiatrists [29]. A detailed psychological intervention plan which was developed in a Chinese hospital to reduce the negative psychological effects that staff at the hospital were experiencing was explained [30]. Included in this programme are leisure activities and training on how to relax and regular visits by psychological counsellors to provide support. 

This study is an advance in learning more about the effects of confinement and especially strict confinement on mental health issues. It is important for taking public health decisions and for implementing procedures by authorities in education establishments to combat the negative effects of confinement on students. Introducing support systems for students could decrease anxiety levels experienced by them in times of confinement and other difficulties. University authorities need to be informed and aware of anxiety suffered by students through research studies so they can improve their support and act rapidly in the future. As a prelude to possible subsequent studies, the effect on students’ academic performance should be analysed. This analysis should consider both a short-term perspective—academic year 2019–2020—and a long-term perspective.

## Figures and Tables

**Table 1:** Demographic and living characteristics.

Characteristic	Number	% Mean (SD)
Age (Mean = 20)	198	33.8 (3.61)
Gender		
Male	65	32.8
Female	133	67.2
Studies		
Business Management	30	15.2
Law and Business	46	23.2
Accounting and Finance	35	17.7
Tourism and Business	19	8.4
Economics	7	3.5
Economics and International Business	57	28.8
Course		
Second	115	58.08
Third	60	30.30
Fourth	23	11.61
Living arrangements		
With parents	187	94.4
Alone or with friends	11	5.6
Family income during COVID-19		
Affected	124	62.6
Not affected	74	37.4
Someone at home infected with COVID-19		
No cases	140	70.7
Confirmed cases	58	29.3
Deaths	17	8.6
Friends/acquaintances infected by COVID-19		
No cases	111	55.9
Confirmed cases	87	43.9
Deaths	13	6.6

**Table 2:** Responses to GAD-7 (7-item Generalized Anxiety Disorder Scale) (*n* = 198).

Anxiety Level	Number	Ratio (%)
Normal	22	11.1
Mild	67	33.8
Moderate	72	36.4
Severe	37	18.7

**Table 3:** GAD-7 and gender (*n* = 198).

Anxiety Level	Gender			
	Male		Female	
	Number	Frequency (%)	Number	Frequency (%)
Normal	14	21.53	8	6.15
Mild	21	32.31	46	34.59
Moderate	22	33.85	50	37.59
Severe	8	12.31	29	21.80
Total	65	100	133	100

**Table 4:** Difficulty to work, to do tasks at home or to be in contact with people.

	No Difficulty	Some Difficulty	Very Difficult	Extremely Difficult	Total
Normal	15	6	1	0	22
Mild	14	41	11	1	67
Moderate	2	43	26	1	72
Severe	0	11	19	7	37
Frequency (%)	15.7	51	28.8	4.5	198

**Table 5:** Factors associated with GAD-7.

Variable	Univariant Analysis		
	Chi-Squared	*p*-Value	Contingency Coefficient
Negatively affected by strict confinement	48.011	<0.001 *	0.442
Relationship before the strict confinement	13.818	0.539	0.255
Relationship during the strict confinement	14.274	0.505	0.259
Feel calm and relaxed	103.761	<0.001 *	0.586
Feel happy and in a good mood	82.125	<0.001 *	0.541
Feel fear	81.418	<0.001 *	0.540
Sleep quality negatively affected	63.501	<0.001 *	0.493
Feel isolated	45.205	<0.001 *	0.431
Living with parents	0.457	0.928	0.048
Satisfaction with the family	37.002	0.005 *	0.397
Family income affected by COVID-19 situation	5.66	0.129	0.167
A family member infected by COVID-19	3.009	0.390	0.112
A friend infected by COVID-19	3.145	0.370	0.125
Death of family member by COVID-19	1.161	0.762	0.076
Death of a friend by COVID-19	1.233	0.745	0.079
How difficult it was to do your job while in lockdown	98.217	<0.001 *	0.576
Continuing with daily habits during this period helps to cope (sports, online classes, personal relationships, reading, etc.)	24.282	0.146	0.331

* Significant variables.

**Table 6:** Factor associated with moderate to severe anxiety during strict confinement.

Variable	Multivariate Logistic Regression Analysis Severe Anxiety R^2^ = 0.546		
	B	Wald	*p*-Value *
Negatively affected by strict confinement	13.33	0.002	<0.001
Feel calm and relaxed (Always)	24.36	0.001	<0.001
To feel fear (Never)	31.52	0.000	0.004
Sleep worse (More than half the time)	7.25	49.37	<0.001
Feeling isolated (Never)	−12.92	0.000	<0.001
Satisfaction with the family (Not satisfied)	30.275	0.000	<0.001
How difficult it has been to do your job while in lockdown (Some difficulty)	5.848	6.689	<0.001
AIC = 444.202 Likelihood = 228.202			

* Level of significance of the model.

## Data Availability

The data presented in this study are available on request from the corresponding author. The data are not publicly available due to being unnecessary for the understanding of the document.

## Supplementary Materials

The following are available online at https://www.mdpi.com/1660-4601/18/4/1710/s1, Table S1: Association between GAD-7 and being affected negatively by the state of alarm by COVID-19 (confinement), Table S2: Association between GAD-7 and immediate family income affected negatively, Table S3: Association between GAD-7 and feeling fear. Table S4: Association between GAD-7 and negatively affected sleep patterns, Table S5: Association between GAD-7 and relationship with family, Table S6: Association between GAD-7 and if their habits help them during this period, Table S7: Association between GAD-7 and feeling calm and relaxed, Table S8: Association between GAD-7 with the death of a relative or a friend.
