# Supplementary material for: The Psychological Impact of Strict and Prolonged Confinement on Business Students during the COVID-19 Pandemic at a Spanish University
Source: Int J Environ Res Public Health. 2021 Feb 10;18(4):1710. doi: 10.3390/ijerph18041710 (PMC7916562; doi:10.3390/ijerph18041710)
Supplement: Supplementary file 1 [file ijerph-18-01710-s001.pdf]

International Journal of
Environmental Research and Public Health

MDPI

Article

The psychological impact of strict and prolonged confinement on business students during the COVID-19 pandemic at a Spanish university

Supplementary Material:

Table S1. Association between GAD-7 and being affected negatively by the state of alarm by Covid-19 (confinement)

Anxiety level	Affected negatively by confinement				
	Yes, a lot	Yes, somewhat	Yes, but only in a small way	No	Total
Normal	5	5	6	6	22
Mild	18	29	17	3	67
Moderate	21	45	5	1	72
Severe	19	16	0	2	37
Total	63	95	28	12	198

Table S2. Association between GAD-7 and immediate family income affected negatively

**Anxiety level**	**Affected**	**Not affected**	**Total**
**Normal**	11	11	22
**Mild**	37	30	67
**Moderate**	49	23	72
**Severe**	27	10	37
**Total**	124	74	198

Table S3. Association between GAD-7 and feeling fear

**Anxiety level**	**Feeling fear**				
	**Always**	**More than 50% of the time**	**Less than 50% of the time**	**Never**	**Total**
**Normal**	0	1	8	13	22
**Mild**	0	17	38	11	67
**Moderate**	1	28	40	3	72

**Severe**	3	19	11	1	37
**Total**	4	65	97	28	198*

*Four values lost

Table S4. Association between GAD-7 and negatively affected sleep patterns

Anxiety level	Negatively affected sleep patterns						
	Always	Most of the time	More than 50% of the time	Less than 50% of the time	Some-times	Never	Total
**Normal**	1	3	1	0	8	9	22
**Mild**	9	20	7	10	12	9	67
**Moderate**	20	21	10	4	10	6	72
**Severe**	20	10	4	3	0	0	37
**Total**	50	54	22	17	30	24	198*

*One value lost

Table S5. Association between GAD-7 and relationship with family

Anxiety level	Level of satisfaction with family relationship						
	Completely satisfied	Very satisfied	Somewhat satisfied	Neither satisfied or nor dissatisfied	Not very satisfied	Not satisfied at all	Total
**Normal**	12	5	3	0	2	0	22
**Mild**	27	29	9	0	1	0	67
**Moderate**	20	27	14	4	6	1	72
**Severe**	7	13	3	4	9	0	37
**Total**	66	74	29	8	18	1	198*

*Two values lost

Table S6. Association between GAD-7 and if their habits help them during this period

Anxiety level	Habits help them during this period						
	Extremely true	True almost always	Somewhat true	Neither true nor false	Somewhat false	False in almost all contexts	Completely false	Total
**Normal**	6	10	4	2	0	0	0	22
**Mild**	17	21	18	6	3	1	1	67
**Moderate**	9	19	25	7	6	5	1	72
**Severe**	2	9	13	5	5	1	2	37
**Total**	34	59	60	20	14	7	4	198

Table S7. Association between GAD-7 and feel calm and relaxed

Anxiety level	Feel calm and relaxed						
	All the time	Most of the time	More than 50% of the time	Less than 50% of the time	Some-times	Never	Total
**Normal**	3	10	4	4	1	0	22
**Mild**	1	9	14	20	21	2	67
**Moderate**	0	3	10	21	33	5	72
**Severe**	0	0	1	4	17	15	37
**Total**	4	22	29	49	72	22	198

Table S8. Association between GAD-7 with the death of a relative or a friend.

Anxiety level	Death of a relative or a friend.					
	Relative			Friend		
	Affected	Not affected	Total	Affected	Not affected	Total
**Normal**	1	21	22	2	20	22
**Mild**	5	62	67	5	62	67
**Moderate**	8	64	72	5	67	72
**Severe**	3	34	37	1	36	37
**Total**	17	181	198	13	185	198
